# The Effects of Early Rehabilitation in the Intensive Care Unit for Patients with Severe COVID-19 Pneumonia: A Retrospective Cohort Study

**DOI:** 10.3390/jcm11020357

**Published:** 2022-01-12

**Authors:** Tokio Kinoshita, Yukihide Nishimura, Yasunori Umemoto, Yasuhisa Fujita, Ken Kouda, Yoshinori Yasuoka, Kyohei Miyamoto, Seiya Kato, Fumihiro Tajima

**Affiliations:** 1 Department of Rehabilitation Medicine, Wakayama Medical University, Wakayama 641-8509, Japan; tokio@wakayama-med.ac.jp (T.K.); r.comet06@gmail.com (Y.U.); y_fujita81@yahoo.co.jp (Y.F.); kenkouda@wakayama-med.ac.jp (K.K.); xjr400yy@yahoo.co.jp (Y.Y.); tajibun@gmail.com (F.T.); 2 Division of Rehabilitation, Wakayama Medical University Hospital, Wakayama 641-8510, Japan; 3 Department of Rehabilitation Medicine, Iwate Medical University, Iwate 020-0023, Japan; 4 Department of Emergency and Critical Care Medicine, Wakayama Medical University, Wakayama 641-8509, Japan; go.go.kyohei.miyamoto@gmail.com (K.M.); katos@wakayama-med.ac.jp (S.K.)

**Keywords:** early ambulation, SARS-CoV-2, adverse events

## Abstract

This retrospective cohort study aimed to examine the rehabilitation effect of patients with coronavirus disease 2019 (COVID-19) in the intensive care unit (ICU) under mechanical ventilation and included ICU patients from a university hospital who received rehabilitation under ventilator control until 31 May 2021. Seven patients were included, and three of them died; thus, the results of the four survivors were examined. The rehabilitation program comprised the extremity range-of-motion training and sitting on the bed’s edge. The Sequential Organ Failure Assessment score (median (25–75th percentiles)) at admission was 7.5 (5.75–8.5), and the activities of daily living (ADLs) were bedridden, the lowest in the Functional Independence Measure (FIM) and Barthel Index (BI) surveys. Data on the mean time to extubation, ICU length of stay, and ADLs improvement (FIM and BI) during ICU admission were obtained. Inferential analyses were not performed considering the small sample size. The mean time to extubation was 4.9 ± 1.1 days, and the ICU length of stay was 11.8 ± 5.0 days. ΔFIM was 36.5 (28.0–40.5), and the ΔBI was 22.5 (3.75–40.0). Moreover, no serious adverse events occurred in the patients during rehabilitation. Early mobilization of patients with COVID-19 may be useful in ADLs improvement during ICU stay.

## 1. Introduction

Currently, there are over 190 million cases of coronavirus disease (COVID-19) infections worldwide, with the number of deaths exceeding 4 million on 20 July 2021 [1]. Approximately 80% of patients have mild diseases, not requiring hospitalization, while the remaining 20% require treatment, with 5% being admitted to the intensive care unit (ICU) and requiring ventilator management [2,3]. In addition, patients often use extracorporeal membrane oxygenation (ECMO).

Patients with severe COVID-19 pneumonia often receive prolonged ventilator management under deep sedation for lung protection. Consequently, ICU-acquired weakness (ICU-AW) and disuse syndrome are likely to occur, resulting in lower activities of daily living (ADL) and longer hospital stays [4,5]. In mid-April 2021, securing hospital rooms for patients with severe COVID-19 became challenging in some Japanese regions, resulting in medical collapse [6].

Early mobilization of ICU patients effectively reduces the duration of intubation and hospital stay and improves ADL [7]. In our hospital, the Department of Emergency and Critical Care Medicine and the Department of Rehabilitation collaborated to implement early mobilization from the early phase of ICU admission, with the same protocol being applied to patients with severe COVID-19 pneumonia [8,9]. However, since COVID-19 is a new disease, there remains scarce information regarding rehabilitation. There are several cautious opinions regarding the implementation of rehabilitation from the perspective of pulmonary protection as early rehabilitation can burden the respiratory system [10].

We hypothesized that early rehabilitation provision is effective even in severely ill patients of COVID-19. This study aimed to provide information for suggesting early rehabilitation programs for patients with COVID-19. Specifically, this study aimed to evaluate the effect of early rehabilitation on patients with COVID-19 pneumonia who underwent ventilator management at the ICU at our hospital and compare the outcomes with the results of previous studies.

## 2. Materials and Methods

### 2.1. Study Design and Setting

This retrospective cohort study was conducted at the Wakayama Medical University Hospital.

### 2.2. Participants

**Figure 1:**
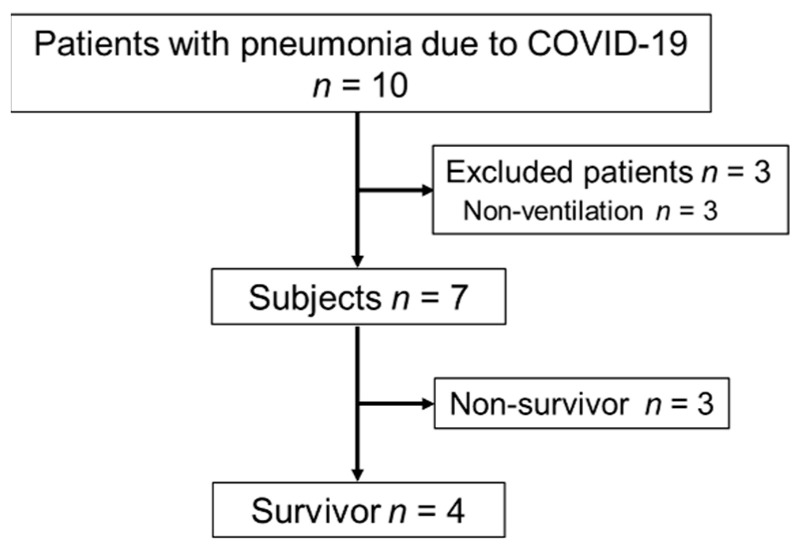
Patients’ flow diagram.

We included patients who were admitted to the ICU of our hospital and received rehabilitation until 31 May 2021. The exclusion criteria were as follows: age < 18 years, low ADL independence (Barthel Index (BI) < 70 points) 2 weeks before admission, and not receiving ventilator management. Figure 1 shows the patients’ flow diagram. In total, 10 patients were managed in the ICU due to COVID-19 pneumonia and received rehabilitation. Among them, we excluded three patients who did not receive ventilator management. Three of the remaining seven patients died, and the other four remaining patients were included in the study. In our hospital, ECMO is introduced when the PaO*_2_*/FiO*_2_* ratio remains below 80 under lung-protective ventilation. However, we excluded cases in which reversibility cannot be expected, such as hypoxemia when the duration of ventilatory management exceeds 1 week. The patients were able to maintain a PaO_2_/FiO_2_ ratio of 80 or higher with the use of prone ventilation and muscle relaxant, so ECMO was not used.

### 2.3. Interventions for Patients with COVID-19

**Figure 2:**
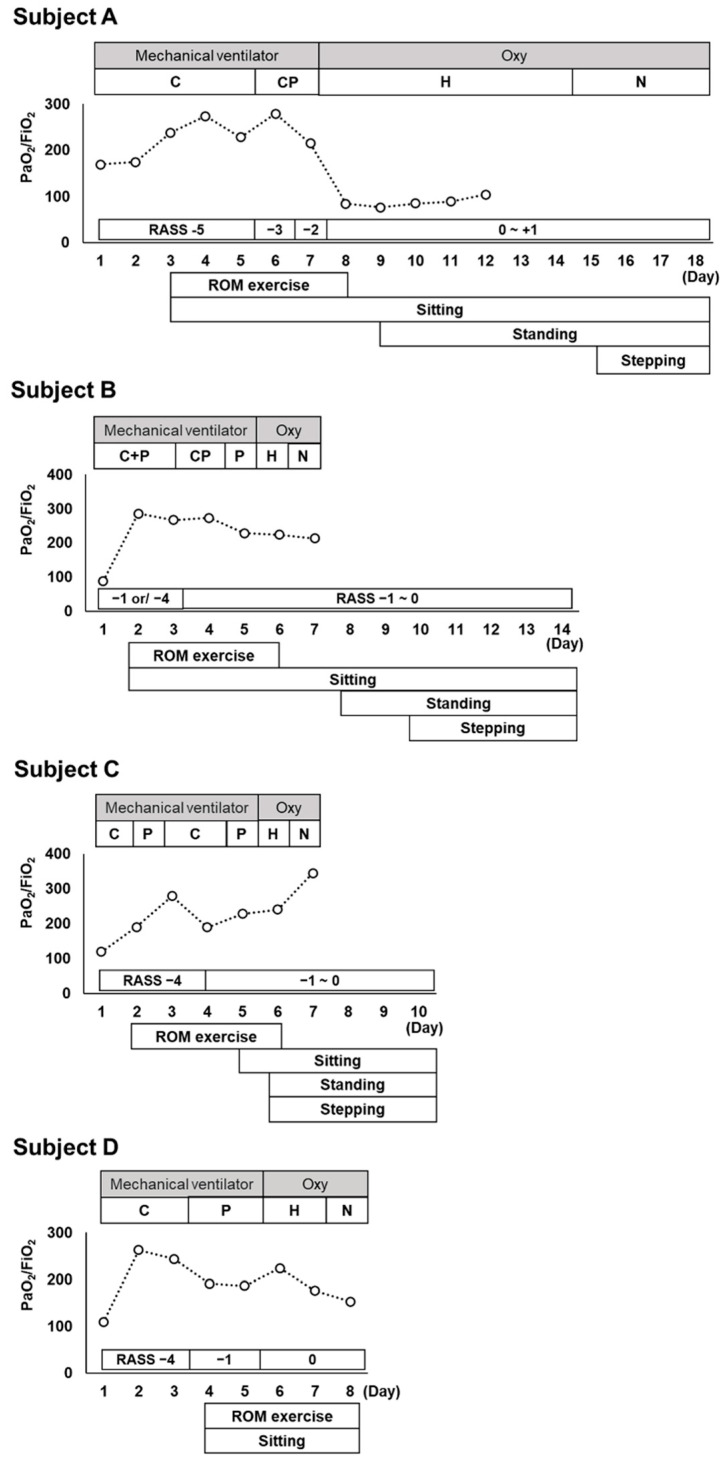
Data recorded during the management of the patients’ pulmonary condition, PaO_2_/FiO_2_ ratio, Sequential Organ Failure Assessment, and rehabilitation programs used during the ICU stay. Respiratory management of subject B for 1–3 days was performed on night C and during the daytime at P. Oxy, oxygen therapy; C, continuous mandatory ventilation (pressure-controlled ventilation); CP, continuous positive airway pressure; H, high-flow nasal cannula; N, nasal cannula; P, pressure support ventilator; RASS, Richmond Agitation–Sedation Scale; ROM, range of motion; sitting, sitting on the edge of the bed.

The university hospital had 10 beds in the ICU. The Department of Emergency and Critical Care Medicine handled particularly severe ICU cases of COVID-19, while moderate-to-mild COVID-19 cases were treated at local hospitals. Rehabilitation began upon request from the intensive care physician to the rehabilitation department. In this hospital, physiatrists examined the patient before rehabilitation onset and evaluated the exact diagnosis, disease state, and physical condition. Subsequently, registered and skilled therapists initiated exercise therapy [11,12]. The initial rehabilitation program comprised extremity passive range-of-motion (ROM) training to prevent extremity contractures, as well as sitting on the edge of the bed to promote sputum evacuation, prevent ICU-AW occurrence, decrease circulating blood volume, and maintain autonomic blood pressure control function. The timing of sitting on the edge of the bed was determined by the intensive care physician based on the respiratory and circulatory status. Figure 2 shows the timeline of each patient’s respiratory management and rehabilitation program.

Subject A was referred to the department of rehabilitation on the day after admission, and consultation with the physiatrist was conducted in the evening. Therefore, the patients were rehabilitated from the third day of admission. The patient was managed under deep sedation with muscle relaxants. Limb ROM training was provided to prevent limited joint mobility of the extremities, with 20 min of sitting on the edge of the bed. On the eighth day of admission, the patient was released from sedation control and was managed with a high-flow nasal cannula; however, oxygenation was poor, and the resting oxygen saturation was low, at approximately 88–92%. Training in the standing position was added on the 9th day, while monitoring the hypoxia, and rehabilitation was provided for approximately 40 min with rest, in addition to the previous training. On the 15th day of admission, the patient was changed to a nasal cannula, and 3 sets of 50 stepping exercises, 20 half squats, and 20 calf raises were added to the training.

Subject B was referred to the department of rehabilitation on the day of admission; however, because it was in the evening, rehabilitation was started the next day. The patient was able to perform simple obedience, and in addition to ROM training, 20 min of sitting on the edge of the bed was performed, wherein sitting could be maintained under the supervision of a therapist. On the third day of admission, the patient was in a delirious state and required constant light assistance in the sitting position, and since the patient could not follow instructions, standing training could not be added. On the eighth day of admission, the delirium was reduced, and the patient was able to follow simple instructions; therefore, standing training was added, and 3 sets of 50 stepping exercises, 20 half squats, and 20 calf raises were added on the 10th day.

Subject C was admitted in the evening and was referred to the department of rehabilitation the next day. The patient was found to have foam sputum, and the intensive care physician instructed only ROM training since he did not wish to increase the airway pressure for lung protection. On the 5th day of admission, the patient’s foam sputum secretion decreased, and 20 min of sitting on the edge of the bed was added. The patient was able to hold the sitting position; therefore, standing, 50 stepping exercises, 20 half squats, and 20 calf raises were added on the 6th day of admission.

Subject D was managed with a 6-L reservoir mask on admission to the ICU but was transferred to ventilatory management owing to hypoxia. Due to the worsening of pneumonia symptoms, no referral to the department of rehabilitation was made on this day. Since the department of rehabilitation was closed on the second and third days of hospitalization, initiation of rehabilitation was delayed until the fourth day. Rehabilitation started with ROM training and 20 min of sitting on the edge of the bed; however, the patient was uncooperative owing to fervent complaints of fatigue. Therefore, we were unable to add standing and stepping training thereafter.

Rehabilitation staff wore personal protective equipment for preventing COVID-19 transmission from wet biological materials and aerosols from patients. To reduce the infection risk, each patient was treated by only one physical therapist and one nurse.

### 2.4. Outcome Measures

The measurement items were the period from intubation to extubation, duration of ICU stay, the extent of ADL improvement during ICU admission, mortality rate, and the number of severe adverse events during rehabilitation. The Functional Independence Measure (FIM) is an 18-item scale for disability severity that comprises the motor (13 items) and cognition (5 items) subscales, which are evaluated on a 7-point ordinal scale [13]. The BI was developed in 1955 as a simple independence index that can provide a disability score. It comprises 10 ADL items with increments of 5 points, with a score of 100 points indicating independence in all 10 items [14]. FIM and BI were measured on admission and on transfer from the ICU. The Sequential Organ Failure Assessment (SOFA) score is a simple and objective score that allows for the calculation of both the number and the severity of organ dysfunction in six organ systems (respiratory, coagulatory, liver, cardiovascular, renal, and neurologic), and the score can measure individual or aggregate organ dysfunction [15]. The Richmond Agitation–Sedation Scale (RASS) was developed in a collaborative effort with practitioners representing critical care physicians, nurses, and pharmacists. RASS is a 10-point scale, with 4 levels of anxiety or agitation (+1 to +4 (combative)), 1 level to denote a calm and alert state (0), and 5 levels of sedation (−1 to −5) culminating in unarousable (−5) [16]. RASS was measured by the nurse before the start of rehabilitation and by the therapist daily after the start of rehabilitation at the time of rehabilitation. Severe adverse events were classified using the National Coordination Council for Medication Error Reporting and Prevention index [17] as F or higher incidents requiring intensive treatment or prolonged hospitalization.

### 2.5. Statistical Analyses

Age, height, weight, body mass index (BMI), duration from ICU admission to start of rehabilitation and to sitting on the edge of the bed, and duration from the start of rehabilitation to death are expressed as mean ± standard deviation. The scores of the SOFA, RASS, FIM, and BI are expressed as median (interquartile range). Given the small sample size, we did not perform inferential analyses.

## 3. Results

Table 1 shows the baseline characteristics of the patients and the duration from ICU admission to starting rehabilitation and sitting on the edge of the bed.

The age of the patients with COVID-19 was 77.0 ± 6.4 years, indicating that the patients were elderly. Moreover, the BMI indicated that the patients were overweight (28.6 ± 6.0 kg/m^2^). The initial SOFA scores of the patients with COVID-19 were 7.5 (5.75–8.5). The RASS score was −4 (−4.25 to −3.5), and the patients were managed under deep sedation. FIM and BI on admission were the lowest, and the patient’s ADL was severely impaired.

The time from intubation to extubation was 4.9 ± 1.1 days. The duration of the ICU stay was 11.8 ± 5.0 days. Further, the ΔFIM was 36.5 (28.0–40.5), and ΔBI was 22.5 (3.75–40.0). (Table 2) The mortality rate of patients with COVID-19 was 42.8%. Furthermore, there were no serious adverse events during rehabilitation. Two of the three who died had been undergoing sitting on the edge of the bed from an early stage after admission, but their condition worsened, and the sitting on the edge of the bed was interrupted, and only ROM training was continued. In addition, one of them had a poor general condition from the time of admission to our hospital, and only ROM training was conducted at the discretion of the intensive care physician. The causes of death in three patients were multiple organ failure in one case and refractory hypoxia due to acute respiratory distress syndrome in two cases.

## 4. Discussion

Early rehabilitation for patients with COVID-19 receiving ventilator management in the ICU appeared to promote ADL improvement, even within the ICU stay. Furthermore, there were no serious adverse events during rehabilitation. The results of this study suggest that early rehabilitation is effective for patients with COVID-19, without imposing a higher risk. To the best of our knowledge, this is the first study to examine the effect of early mobilization in patients with COVID-19 pneumonia; moreover, our findings could inform early rehabilitation for patients with COVID-19.

Early mobilization of ICU patients effectively reduces the time to extubation and duration of hospital stay and improves the ADL [7]. Additionally, early rehabilitation has been recently recommended for patients with COVID-19 in the ICU [5,8,9]. Despite numerous concerns regarding the rehabilitation of patients with severe COVID-19 pneumonia, a consensus has been reached on several fronts, including respiratory training, moderate head elevation, limb mobilization, and sitting and standing positions at the bedside [18]. Our findings encourage the use of early rehabilitation. Contrastingly, in COVID-19 pneumonia, increased spontaneous breathing may decrease the intrathoracic pressure, which causes pulmonary edema and worsens the respiratory status [19]. Local forces generated by respiratory muscles in patients with pre-existing lung injury may cause local injurious effects. Additionally, the increased transmural pulmonary vascular pressure amplitude produced by inspiratory effort may exacerbate vascular leakage [20]. Respiratory management of patients with severe COVID-19 is based on lung protection, which is consistent with the treatment of acute respiratory distress syndrome, involving low tidal volume, low driving pressure, the addition of positive end-expiratory pressure, and long-term ventilator management [21]. This could increase the duration until extubation. In a previous study, wherein early positioning and other chest physiotherapy were provided to patients with COVID-19 admitted to the ICU, the time to extubation was 19 ± 10 days [22], which is longer than that at our hospital (4.9 ± 1.1 days). Therefore, early mobilization of patients with COVID-19 may have fewer adverse effects on the respiratory system. Furthermore, the duration of ICU stay in this previous study was 22 ± 11 days [22], whereas that in our study was 11.8 ± 5.0 days. Initially, our hospital lacked beds for moderately symptomatic patients since only patients with severe COVID-19 were treated. Therefore, even for patients with improved respiratory conditions, it was difficult to secure a bed for transfer from the ICU. Under normal conditions, patients were rarely admitted to the ICU under nasal cannula management, which could have underestimated the findings of the COVID-19 group. Early rehabilitation of patients with COVID-19 appeared to effectively reduce the time to extubation and duration of ICU stay. However, it is difficult to make a direct comparison because the criteria for extubation and ICU discharge varied among the facilities, indicating the need for further investigation.

A previous study demonstrated the results of the BI of the patients of COVID-19 at transfer from the ICU and when admitted to the ICU (mean (SEM)) was 10.25 (1.29) [23]. In contrast, the BI at ICU transfer in this study was 21.25 (2), which was approximately twice as high. It has been reported that ICU patients undergoing rehabilitation within 24 h have more significant improvement in the FIM score and walking ability, compared with those who start rehabilitation later [7]. Further, within one day of lying down, the plasma volume, muscle mass, and muscle strength decreased by 5–15% [24], 0.5%, and 0.3–4.2% [25], respectively. The difference in the start time of sitting on the edge of the bed may have affected our findings. In the previous study, only half of the patients received rehabilitation, and the timing of starting the rehabilitation and early mobilization was not mentioned [23]. However, our rehabilitation program, in which patients were mobilized as early as possible, may be effective in improving the ADL of patients with COVID-19.

Previous studies have reported no adverse events requiring additional treatment, increased costs, or extended hospital stay during the rehabilitation of ICU patients [26,27,28], which is consistent with our findings. However, three of seven ICU patients who received rehabilitation under ventilator management died. Previous studies have suggested that early rehabilitation in the ICU does not increase mortality [28,29]. Among the three patients who died, one was rehabilitated only with passive ROM exercises due to poor circulatory and respiratory statuses. Additionally, the other two patients were asked to sit on the edge of the bed while checking their circulatory and respiratory status. However, the patients’ condition did not deteriorate after implementing rehabilitation. In addition, the duration from the start of rehabilitation to death was approximately 20 days; therefore, we believe the start of early rehabilitation was unrelated to death. Although non-survivor patients in this study were younger than the survivors and had a BMI in the standard range, their median SOFA score was higher than that of the survivors. A previous study reported mortality rates of 50%, 95% in the ICU patients with an initial SOFA score of 10, ≥ 11, respectively [30]. Our hospital only treats patients with severe COVID-19. The SOFA scores of the three patients who died were 10, 10, and 14. The increased mortality rate in patients with COVID-19 could be attributed to severe multiple organ failure upon ICU admission. Therefore, the provision of rehabilitation did not seem to affect the mortality rate.

## 5. Limitations

Owing to the small number of patients with COVID-19 admitted to our hospital, we were not able to examine the effect of early rehabilitation or no rehabilitation. Furthermore, it is difficult to generalize the results of this study because the survey was conducted at a single facility. In the future, it is critical to conduct large-scale surveys across multiple facilities.

## 6. Conclusions

Patients with severe COVID-19 requiring ventilation are prone to muscle weakness and exercise intolerance. Therefore, early rehabilitation in the acute disease stage is essential for improving their physical functions. Our findings suggest that patients with COVID-19 pneumonia might need rehabilitation from the early disease stage. Early rehabilitation may be one of the major strategies for preventing medical collapse.

## Figures and Tables

**Table 1:** Baseline patient characteristics and duration from ICU admission to starting rehabilitation and sitting.

	COVID-19 Pneumonia (*n* = 4)	Non-Survivor (*n* = 3)
Age (years)	77.0 ± 6.4	70.3 ± 7.5
Sex (females/males)	2/2	1/2
Height (cm)	160.8 ± 10.4	165.8 ± 14.1
Weight (kg)	73.2 ± 12.4	64.1 ± 12.3
Body mass index (kg/m^2^)	28.6 ± 6.0	23.2 ± 1.5
Initial SOFA score	7.5 (5.75–8.5)	10.0 (10.0–12.0)
Initial RASS score	−4 (−4.25–−3.5)	−4 (−4–−4)
Initial total FIM score	18 (18–20.5)	18 (18–18)
Initial total BI	0 (0–0)	0 (0–0)
Duration from ICU admission to starting rehabilitation (h)	40.0 ± 25.8	111.7 ± 150.1
Duration from ICU admission to starting to sit on the edge of the bed (h)	63.0 ± 31.3	Not application ^(a)^
Duration from start of rehabilitation to death (h)	Not applicable	475.3 ± 394.3

Abbreviations: SOFA, Sequential Organ Failure Assessment; RASS, Richmond Agitation–Sedation Scale; FIM, Functional Independence Measure; BI, Barthel Index; ICU, intensive care unit. The initial scores of the SOFA, RASS, FIM, and BI are shown as medians (25th–75th percentiles). ^(a)^ Only 2 of the 4 patients could perform end-sitting training.

**Table 2:** Duration of intubation and ICU stay and the ΔFIM and ΔBI.

	COVID-19 Pneumonia (*n* = 4)
Duration of intubation (day)	4.9 ± 1.1
Duration of ICU stay (day)	11.8 ± 5.0
ΔFIM	36.5 (28.0–40.5)
ΔBI	22.5 (3.75–40.0)

Abbreviations: ICU, intensive care unit; FIM, Functional Independence Measure; BI, Barthel Index. ΔFIM and BI show the extent of improvement from ICU admission to discharge. FIM and BI are shown as median (25–75th percentiles).

## Data Availability

The datasets used and/or analyzed during this study are available from the corresponding author upon reasonable request.

## References

[1] World Health Organization Weekly Epidemiological Update on COVID-19. https://www.who.int/publications/m/item/weekly-epidemiological-update-on-covid-19---20-july-2021.

[2] Guan W.-J., Ni Z.-Y., Hu Y., Liang W.-H., Ou C.-Q., He J.-X., Liu L., Shan H., Lei C.-L., Hui D.S.C. (2020). China Medical Treatment Expert Group for Covid. Clinical characteristics of coronavirus disease 2019 in China. N. Engl. J. Med..

[3] Wu Z., McGoogan J.M. (2020). Characteristics of and important lessons from the coronavirus Disease 2019 (COVID-19) outbreak in China: Summary of a report of 72,314 cases from the Chinese Center for Disease Control and Prevention. JAMA.

[4] Kress J.P., Hall J.B. (2014). ICU-acquired weakness and recovery from critical illness. N. Engl. J. Med..

[5] Thomas P., Baldwin C., Bissett B., Boden I., Gosselink R., Granger C.L., Hodgson C., Jones A.Y., Kho M.E., Moses R. (2020). Physiotherapy management for COVID-19 in the acute hospital setting: Clinical practice recommendations. J. Physiother..

[6] Osaka Prefectural Govemment: Materials from the 47th meeting of the Osaka Prefecture Task Force on Novel Coronaviruses (In Japanese). https://www.pref.osaka.lg.jp/attach/38215/00393224/re-ikkatsu-47.pdf.

[7] Schweickert W.D., Pohlman M.C., Pohlman A.S., Nigos C., Pawlik A.J., Esbrook C.L., Spears L., Miller M., Franczyk M., Deprizio D. (2009). Early physical and occupational therapy in mechanically ventilated, critically ill patients: A randomised controlled trial. Lancet.

[8] Kinoshita T., Umemoto Y., Yasuoka Y., Yoshikawa T., Kouda K., Hori S., Mikami Y., Nishimura Y., Miyamoto K., Kato S. (2021). Feasibility of sit training for patients with severe COVID-19 pneumonia during deep sedation: A case report. Medicine.

[9] Kinoshita T., Kouda K., Umemoto Y., Yasuoka Y., Minoshima Y., Mikami Y., Nishimura Y., Miyamoto K., Kato S., Tajima F. (2021). Case report: A rehabilitation practice report during ICU management for a patient with multiple disabilities due to COVID-19 pneumonia and COPD. Front Med..

[10] Vitacca M., Carone M., Clini E.M., Paneroni M., Lazzeri M., Lanza A., Privitera E., Pasqua F., Gigliotti F., Castellana G. (2020). Joint statement on the role of respiratory rehabilitation in the COVID-19 crisis: The Italian position paper. Respiration.

[11] Kinoshita T., Nishimura Y., Nakamura T., Hashizaki T., Kojima D., Kawanishi M., Uenishi H., Arakawa H., Ogawa T., Kamijo Y.-I. (2017). Effects of physiatrist and registered therapist operating acute rehabilitation (PROr) in patients with stroke. PLoS ONE.

[12] Kinoshita T., Yoshikawa T., Nishimura Y., Kamijo Y.-I., Arakawa H., Nakamura T., Hashizaki T., Hoekstra S.P., Tajima F. (2020). Mobilization within 24 hours of new-onset stroke enhances the rate of home discharge at 6-months follow-up: A prospective cohort study. Int. J. Neurosci..

[13] Keith R.A., Granger C.V., Hamilton B.B., Sherwin F.S. (1987). The Functional Independence Measure: A new tool for rehabilitation. Adv. Clin. Rehabil..

[14] Mahoney F.I., Barthel D.W. (1965). Functional evaluation: The Barthel index. Md. State Med. J..

[15] Vincent J.L., Moreno R., Takala J., Willatts S., De Mendonça A., Bruining H., Reinhart C.K., Suter P.M., Thijs L.G. (1996). The SOFA (Sepsis-related Organ Failure Assessment) score to describe organ dysfunction/failure. On behalf of the Working Group on Sepsis-Related Problems of the European Society of Intensive Care Medicine. Intensive Care Med..

[16] Sessler C.N., Gosnell M.S., Grap M.J., Brophy G.M., O’Neal P.V., Keane K.A., Tesoro E.P., Elswick R.K. (2002). The Richmond Agitation-Sedation Scale: Validity and reliability in adult intensive care unit patients. Am. J. Respir. Crit. Care Med..

[17] National Coordinating Council for Medication Error Reporting and Prevention (2001). NCC Merp Index For Categorizing Medication Errors. https://www.nccmerp.org/sites/default/files/indexBW2001-06-12.pdf.

[18] Li J. (2020). Rehabilitation management of patients with COVID-19: Lessons learned from the first experience in China. Eur. J. Phys. Rehabil. Med..

[19] Gattinoni L., Chiumello D., Caironi P., Busana M., Romitti F., Brazzi L., Camporota L. (2020). COVID-19 pneumonia: Different respiratory treatments for different phenotypes?. Intensive Care Med..

[20] Brochard L., Slutsky A., Pesenti A. (2017). Mechanical ventilation to minimize progression of lung injury in acute respiratory failure. Am. J. Respir. Crit. Care Med..

[21] Ferguson N.D., Fan E., Camporota L., Antonelli M., Anzueto A., Beale R., Brochard L., Brower R., Esteban A., Gattinoni L. (2012). The Berlin definition of ARDS: An expanded rationale, justification, and supplementary material. Intensive Care Med..

[22] McWilliams D., Weblin J., Hodson J., Veenith T., Whitehouse T., Snelson C. (2021). Rehabilitation Levels in Patients with COVID-19 Admitted to Intensive Care Requiring Invasive Ventilation. An Observational Study. Ann. Am. Thorac. Soc..

[23] Musheyev B., Borg L., Janowicz R., Matarlo M., Boyle H., Singh G., Ende V., Babatsikos I., Hou W., Duong T.Q. (2021). Functional status of mechanically ventilated COVID-19 survivors at ICU and hospital discharge. J. Intensive Care.

[24] Greenleaf J.E. (1984). Physiological responses to prolonged bed rest and fluid immersion in humans. J. Appl. Physiol. Respir. Environ. Exerc. Physiol..

[25] Wall B.T., van Loon L.J. (2013). Nutritional strategies to attenuate muscle disuse atrophy. Nutr. Rev..

[26] Lee H., Ko Y.J., Suh G.Y., Yang J.H., Park C.-M., Jeon K., Park Y.H., Chung C.R. (2015). Safety profile and feasibility of early physical therapy and mobility for critically ill patients in the medical intensive care unit: Beginning experiences in Korea. J. Crit. Care.

[27] Sricharoenchai T., Parker A.M., Zanni J.M., Nelliot A., Dinglas V.D., Needham D.M. (2014). Safety of physical therapy interventions in critically ill patients: A single-center prospective evaluation of 1110 intensive care unit admissions. J. Crit. Care.

[28] Zhang L., Hu W., Cai Z., Liu J., Wu J., Deng Y., Yu K., Chen X., Zhu L., Ma J. (2019). Early mobilization of critically ill patients in the intensive care unit: A systematic review and meta-analysis. PLoS ONE.

[29] Okada Y., Unoki T., Matsuishi Y., Egawa Y., Hayashida K., Inoue S. (2019). Early versus delayed mobilization for in-hospital mortality and health-related quality of life among critically ill patients: A systematic review and meta-analysis. J. Intensive Care.

[30] Ferreira F.L., Bota D.P., Bross A., Mélot C., Vincent J.L. (2001). Serial evaluation of the SOFA score to predict outcome in critically ill patients. JAMA.

